# Equation of State of Autoclaved Aerated Concrete–Oedometric Testing

**DOI:** 10.3390/ma17040956

**Published:** 2024-02-19

**Authors:** Yuri S. Karinski, Vladimir R. Feldgun, David Z. Yankelevsky

**Affiliations:** 1 National Building Research Institute, Technion, Haifa 3200003, Israel; aefeldgo@technion.ac.il (V.R.F.); davidyri@technion.ac.il (D.Z.Y.); 2 Faculty of Civil & Environmental Engineering, Technion, Haifa 3200003, Israel

**Keywords:** equation of state, autoclaved aerated concrete, oedometric test, energy dissipation

## Abstract

This paper aims at investigating the triaxial behavior of Autoclaved Aerated Concrete (AAC) under extremely high pressures, and experimentally determine Equation of State (EOS) for several different AAC densities. Oedometric tests were carried out using a home-made high-pressure triaxial apparatus, and pressures up to ~500 MPa were applied. The complete pressure-bulk strain relationships were measured, and new findings and insights were obtained. The paper presents the testing set-up and the measurement system. The data processing method accounting for the AAC pronounced shortening during the ongoing test is described using a weighted functions procedure for the circumferential strains’ calculation, with which the confining pressure was determined. The boundary conditions effects on the test results were investigated, and a new technique for specimen insulation was suggested to ensure loading without friction and the prevention of local shear failure. The experimental EOS for different AAC densities were obtained. EOS curves for different specimens with the same density demonstrated good to very good repeatability of the EOS curves over the entire pressure range. Based on the tests results and the density’s span, three classes of AAC are proposed. A preliminary attempt to apply the newly obtained EOS curves has been carried out to examine the energy dissipation for three different dynamic load levels. Although this is a preliminary stage that is beyond the objective of this paper, early interesting results were observed where an optimal AAC density, for which the highest energy has been absorbed, was identified. This finding encourages inclusion of that preliminary study as a closure section. Numerical simulations of wave propagation through ACC layers of different densities, laid on rigid supporting slabs, was carried out. The minimum total impulse imparted to the rigid slab was found for the optimal AAC density that has been determined above.

## 1. Introduction

Autoclaved aerated concrete (AAC) is a concrete building material that is used for production of lightweight cellular prefabricated masonry blocks and walls. These elements are known for their enhanced thermal and acoustic insulation properties [1], fire resistance, light weight (low density), and high compressibility under pressure [2], thus absorbing plastic energy. Stress–strain behavior of cellular materials exhibits a unique non-linear deformation behavior. Different cellular materials, other than AAC, are used in various applications such as insulation, sandwich panels cores, various automotive parts, as fillers of light-weight structural elements, packaging, and, more recently, in different armor solutions [3,4,5,6,7,8,9,10,11,12,13]. In contrast to metals that commonly maintain constant volume during plastic deformation, cellular materials undergo large volume changes during compressive loading due to buckling and collapse of the interior thin partition walls between cells.

AAC is considerably lighter than concrete. Its dry bulk density is ~250–800 kg/m^3^, which is 10%–30% of ordinary concrete density. The uniaxial compressive strength of AAC blocks increases with the density and ranges between ~2–8 MPa [14,15,16]. The combination of relatively low thermal conductivity and the load bearing capacity is often mentioned as an advantage of AAC compared to metallic foams, for which they are widely used in structural applications [1] in the construction of residential, commercial, and industrial buildings [16,17]. The AAC blocks are mainly used in infill masonry walls [1,18,19] and for sandwich concrete elements [20,21,22,23,24]. These applications of masonry blocks are associated with low magnitude compression, tension, and shear stresses. However, the pronounced compressibility of AAC under high compressive stresses, that is accompanied by large plastic deformation of the AAC material, has been hardly investigated. That behavior may be exploited to provide resistance and energy absorption capacity to high intensity dynamic loads, such as impact loads and high magnitude blast pressures [25,26]. While other cellular materials that differ from AAC, such as aluminum foam [5,6,27,28,29] or foam concrete [30,31,32,33,34], have gained relatively more attention for these implementations, the response of AAC to high dynamic effects and its energy absorption characteristics have gained rather little attention [20,21,22,35].

A limited number of studies were carried out on the constitutive behavior of AAC [21,22] including its EOS [36]. The EOS is an important feature of the cellular material, exhibiting the relationship between the hydrostatic pressure applied on a specimen and the resulting volumetric strain. The EOS has a major role in the impact response of a porous material element. Most of the previous studies examined small AAC specimens using a split Hopkinson bar apparatus. Different ACC types were examined [37], showing a significant scatter of the results and indicating a strong size effect. 

The limited number of studies on AAC in that regard motivate for a somewhat extended literature survey of other cellular materials. It is interesting to note that, for both aluminum foam and AAC, most of experimental output (except [36]) includes the axial “stress–strain” relationships, which may be used for simplified analyses of one-dimensional problems. In the general case of three-dimensional analyses, the equation of state (i.e., the “hydrostatic pressure-bulk strain”) is necessary. Extending the survey to foam concrete, more publications in which the above stress–strain dependences are obtained [31,33,38,39,40,41,42,43,44,45], most of which present uniaxial stress–strain relationships. Reference [45] may be highlighted, as it presents experiments on foamed concrete specimens under uniaxial and triaxial conditions. Similar uniaxial and triaxial experiments were presented on cellular concrete in [46]. 

That limited state of knowledge requires further investigation on the EOS of AAC. The present study aims at an extensive experimental investigation, thus extending the current state of the art in the following aspects: the pressure-density relationship is studied to extremely high pressures, where the specimen undergoes extremely large volumetric strain; a wide range of AAC densities is examined, covering the entire practical range; and relatively large specimens are tested which require high-capacity apparatus, that provide more representative results, as the larger diameter specimens are less affected by peripheral effects.

The experimental quasi-static EOS of cementitious materials may be obtained from tri-axial hydrostatic tests [47,48,49] or uniaxial confined (oedometric [47,50,51,52,53]) tests. The oedometric test technique that is based on uniaxial strain conditions in the tested specimens is more affordable and is technically simpler. The oedometric test has been proven to provide quality results at a large pressure range up to ~1 GPa [48,49,50,51,52]. Note that although the EOS is produced by quasi-static loading, it may be implemented for dynamic analysis and used in “hydro-codes”, as is widely done in different commercial software (i.e., ANSYS AUTODIN [54] and LS-DYNA [55]), where strain-rate effect is introduced in the deviatoric component, while the EOS does not exhibit a strain-rate effect (i.e., P-a model [56], that is recommended for cementitious materials).

This paper aims at an experimental study of the EOS for different AAC densities and examine what is the optimal density in terms of its maximum energy absorption. The oedometric tests have been performed using a home-made high-pressure apparatus [50,51,52]. In these specific tests, a controlled axial (vertical) strain dominates the strain field in the tested specimens and varies within a very large range of the bulk strains. To follow the lateral stress during these large displacements process, a special procedure of hoop strain calculations is developed. 

## 2. Testing Preparations

### 2.1. The Test Specimens—General Description

AAC ~50 × 25 × 30 cm masonry blocks were produced at an industrial plant (Ytong, Pardes Hanna-Karkur, Israel) under the plant quality-controlled supervision, aiming at preparing blocks with four different density levels within the common practical demand. The densities within each group showed good repeatability. To avoid the possible density difference between blocks having a similar density level (AAC type), cylindrical cores for all specimens representing a certain density level were taken from a single block. The oedometric behavior of the AAC specimens with the four different initial densities was investigated. The specimens were classified and identified by their densities level as follows (see also Table 1): D660 ~630–690 kg/m^3^; D600 ~595–610 kg/m^3^; D400 ~403–430 kg/m^3^; and D280 ~280–300 kg/m^3^.

**Figure 1:**
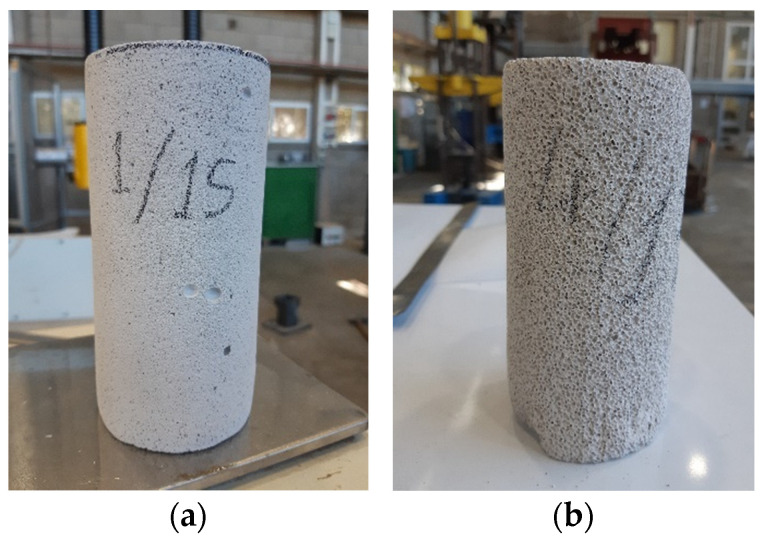
View of different specimens. (**a**) Highest density. (**b**) Lowest density.

Four specimens were prepared for each density, and extra specimens (three specimens of the highest density, D660, and one specimen of the lowest density D280) were prepared for further specific testing, aiming at examining the effects of boundary conditions (see Section 4.2). The specimens were produced by wet drilling from the blocks. The drilled specimens were examined to eliminate specimens with any observed damage and then were dried in a climate-controlled room. The dimensions of all specimens were 139.5 ± 1.4 mm in height and 68 ± 1 mm in diameter (Figure 1a,b). The notations and data of each specimen are given in Table 1. It shows a small variability of the specimens’ densities with a small Coefficient of Variation (CoV).

### 2.2. Test Setup

**Figure 2:**
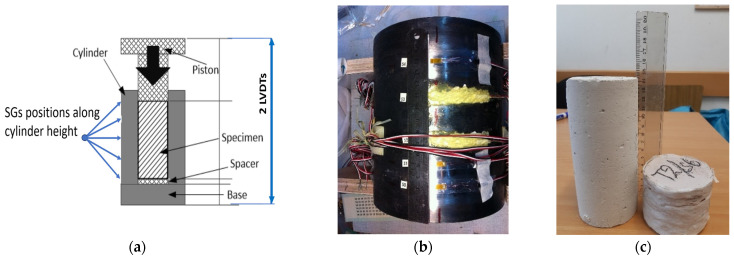
Test set-up and specimen. (**a**) The test set-up scheme. (**b**) SGs on cylinder face. (**c**) Left: Original specimen; right: compressed specimen.

**Figure 3:**
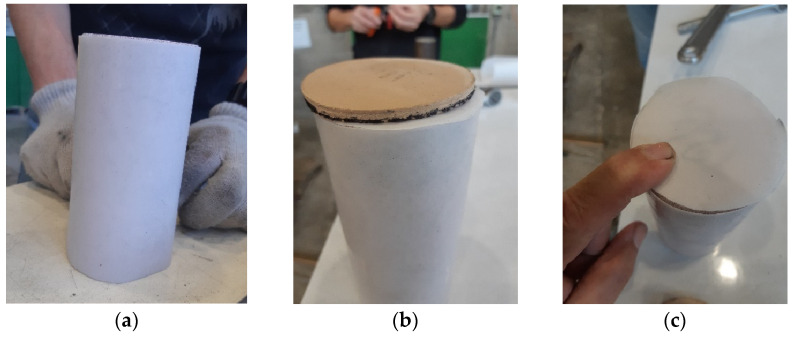
Specimen insulation. (**a**) Cylinder wrapping. (**b**) Rubber disc on top face. (**c**) Top Teflon disc.

The home-made test set-up is presented in detail in [50,51,52]. It includes a high-strength steel thick-walled hollow cylinder with a 70 mm diameter borehole (Figure 2a) that is placed on a thick steel base. The outer cylinder surface is instrumented with 15 strain gauges (SGs – MM, PA, USA). The SGs are placed at five levels along the cylinder height (Figure 2a), three SGs at each level, installed at equal sector angles (120°). The SGs aim at measuring the hoop strain on the outer thick cylinder surface. A 14.5-mm-thick, high-strength steel disc (denoted “the spacer”), having the borehole diameter, is placed at the borehole bottom over the thick-base top face. It aims at controlling the specimen vertical position along the borehole height. A 0.2-mm-thick, 70 mm diameter Teflon disc is placed on top of the spacer to prevent friction. A 5-mm-thick, 71 mm diameter circular rubber disc is pressed against the Teflon disc to seal the borehole bottom and allow evenly distributed pressure on the specimen’s bottom face, thus avoiding local stress concentration and the resulting possible damage. The test specimen is wrapped by a thin Teflon film (Figure 3a) to avoid friction with the borehole inner face [57]. It is then pushed into the borehole until reaching the rubber disc. A similar rubber disc is then pressed against the specimen top face, and another thin Teflon disc is inserted and placed on top of it (Figure 3b,c).

A long steel piston is then inserted into the cylinder borehole until its bottom maintains contact with the top Teflon disc (Figure 2a).

### 2.3. Testing Procedure

The test setup, including the installed specimen, is mounted in the loading frame and connected to the 5000 kN loading jack. The vertical load is measured by a 5000-kN-capacity load cell (HBM, Germany). The total axial displacement is measured using two linear variable displacement transducers (LVDT’s, Measurement Specialties, Shenzhen, China) measuring the total displacement between the piston-loaded top face and the bottom of the cylinder base plate (Figure 2a). The measured hoop strain on the exterior face of the steel cylinder is used to calculate a lateral confining pressure acting on the specimen during the test and radially outwards on the cylinder-thick wall. In the case of oedometric tests of concrete specimens, the axial displacement of a specimen is relatively small due to the specimen’s very high stiffness. In this case, all SGs along the cylinder height sense the direct effect of the confining pressure. In the present case of oedometric tests on AAC specimens, very large axial displacements and strains are developed (Figure 2c), reducing the specimen height by up to ~80%. 

This poses a unique problem that should be addressed. During testing, the AAC specimen height decreases considerably, and finally it faces only the lower level SGs. Hence, to allow more accurate evaluation of the confining pressure acting against the cylinder’s wall over a limited part of its height, a special procedure is required to account for the participating SGs at each stage of loading. This procedure for the lateral pressure’s calculation is described in Section 3.2 below.

The loading is performed at a low quasi-static rate of ~5 kN/s using a force-control actuator (Alfred J. Amsler & Co., Schaffhouse, Switzerland). The loading is accompanied by monitoring the load, total displacement, and the SGs readings every 1 s.

Four specimens of each density were tested under the specified above boundary conditions. To check the effect of the boundary conditions, two additional specimens (of the highest and lowest densities) were tested under different boundary conditions (see Section 4.2). 

## 3. Testing Program

### 3.1. Preliminary Tests

**Figure 4:**
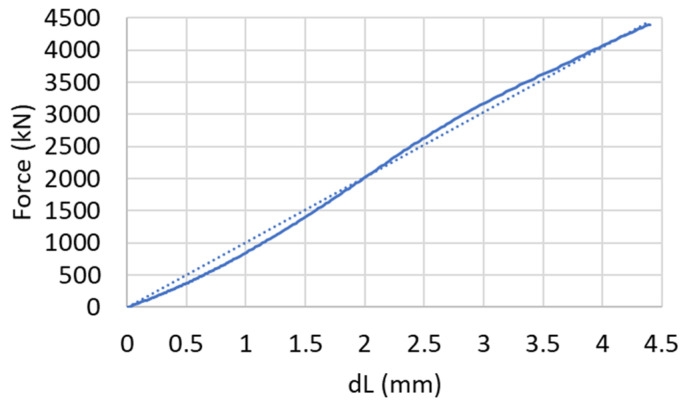
Compression of rubber discs and steel component: measured (solid) and approximated (dotted) curves.

The vertical displacement of the piston, *Dl*, that is measured by two LVDT’s, yields the total displacement due to the applied vertical compression. That displacement is the sum of three different material components that are laid in series: (1) the steel components, including the piston, spacer and the steel base; (2) the two intermediate rubber discs; (3) and an the AAC specimen. All three components are subjected to the same axial load. To determine the load–displacement relationship of the specimen, the force-displacement relationships of the steel and rubber components should be determined a priori and eliminated from the total load–displacement relationship that is measured by the LVDTs. For that purpose, two rubber discs (total height of 10 mm) were placed over the base plate at the borehole bottom, and the steel piston was inserted into the borehole until reaching contact with these discs. The loading of that system yields the load–displacement relationship of the steel and rubber components, which is depicted in Figure 4. The measured curve (Figure 4, solid line) shows an almost linear force-displacement relationship, with a 40 mm displacement at a compressive load of ~4000 kN. The linear approximate relationship (*R*^2^ = 0.9974) may be adopted for further analysis (Figure 4, dotted line). The corresponding constant stiffness of that curve is k = 101.35 kN/mm.

Hence, the specimen axial strain (vertical direction) is calculated from:

(1) $$\varepsilon_{z}=\frac{\left(∆l-F/k\right)}{l_{0}}$$

where:

*l*_0_—is the specimen height;
*F*—is the applied force.

### 3.2. Lateral Pressure Calculation

The confining (lateral) pressure acting on the specimen was calculated based on the elastic response of the thick cylinder that is subjected to the unknown evenly distributed inner pressure. The inner pressure is producing the cylinder circumferential extension that is measured by the SGs. 

For the given tests, the highest level SGs (“54” in Figure 2a) and lowest level SGs (“50” in Figure 2a) are located at the levels of the top and bottom rubber discs, and their records are not considered. At the beginning of the test, all three “central” levels of SG’s (51, 52, 53 in Figure 2a) measure the hoop strain correctly at the beginning of the test. During the test, the level of the specimen top surface lowers gradually towards the level of SGs “54” and then towards the level of SGs “53” (Figure 2a) and beyond, as may be observed from the highest density specimen, D660, before and after the test (Figure 2a). That means that, at first, the test top SG’s level, and then the middle level, are no longer in contact with the specimen, and their measurements are no longer directly related to the lateral pressure acting on the specimen and should be omitted from the analysis. Therefore, some weighted functions, *w_up_*, *w_mid_*, and *w_down_*, are required. In the present study, the piecewise linear weighted functions have been implemented, as presented in Equation (3). The indexes “up”, “mid”, and “down” refer to strain gauges levels “53”, “52”, and “51” in Figure 2b, respectively. The functions are dependent on the measured displacement ${∆l}_{0}$ and on the SGs coordinate (relative to the cylinder’s top). Finally, the hoop strain is calculated as follows:

(2) $$\varepsilon_{hoop}={w_{up}\cdot \varepsilon }_{up}+w_{mid}\cdot \varepsilon_{mid}+{w_{down}\cdot \varepsilon }_{down}$$

(3) $$\begin{array}{c} w_{up}=\left\{\begin{array}{cc} \frac{h_{up}-{∆l}_{0}}{3h_{up}} & {{∆l}_{0}\leq h}_{up}\\ 0 & {{∆l}_{0}>h}_{up} \end{array}\right.\;;\;w_{mid}=\left\{\begin{array}{cc} \frac{1}{3}+\frac{{∆l}_{0}}{6h_{up}} & {{∆l}_{0}\leq h}_{up}\\ \frac{h_{mid}-{∆l}_{0}}{2\left({h_{mid}-h}_{up}\right)} & h_{up}<{{∆l}_{0}\leq h}_{mid}\\ 0 & {{∆l}_{0}>h}_{mid} \end{array}\right.;\\ w_{down}=1-w_{mid}-w_{up} \end{array}$$

where ${∆l}_{0}=(∆l-F/k)$; *e_up_*, *e_mid_*, and *e_down_* are the averages of three SGs measurements at the corresponding levels “53”, “52” and “51”, respectively (Figure 2a); and *h_up_* and *h_mid_* are the distance of the “53” and “52” SG levels from the top point of the cylinder (70 and 110 mm, respectively).

The lateral pressure is calculated as follows: $\sigma_{r}=\lambda \varepsilon_{hoop}$, where *l*~0.38 MPa/mstrain is a lateral pressure coefficient that had been obtained in [51,52] from interior hydrostatic pressure loading in the cylinder borehole, carried out using hydraulic grease. Note that the factor *l* is the only property of the steel device’s cylinder, and does not depend on the specimen shortening. 

Hydrostatic pressure, *p*, and bulk strain, *e_V_*, are calculated as follows:

(4) $$p=\frac{1}{3}\left(\frac{4F}{\pi {rd}_{0}^{2}}+2\lambda \varepsilon_{hoop}\right);\;\;\;\;\;\;\varepsilon_{V}=\varepsilon_{z}+\varepsilon_{hoop}\;\left(\left(1-\nu \right)+(1+\nu )\frac{D_{0}^{2}}{d_{0}^{2}}\right)$$

where *d*_0_ is the specimen diameter (Table 1) and *D*_0_ is the outer diameter of the cylinder.

## 4. Experimental Results

### 4.1. Repeatability

**Figure 5:**
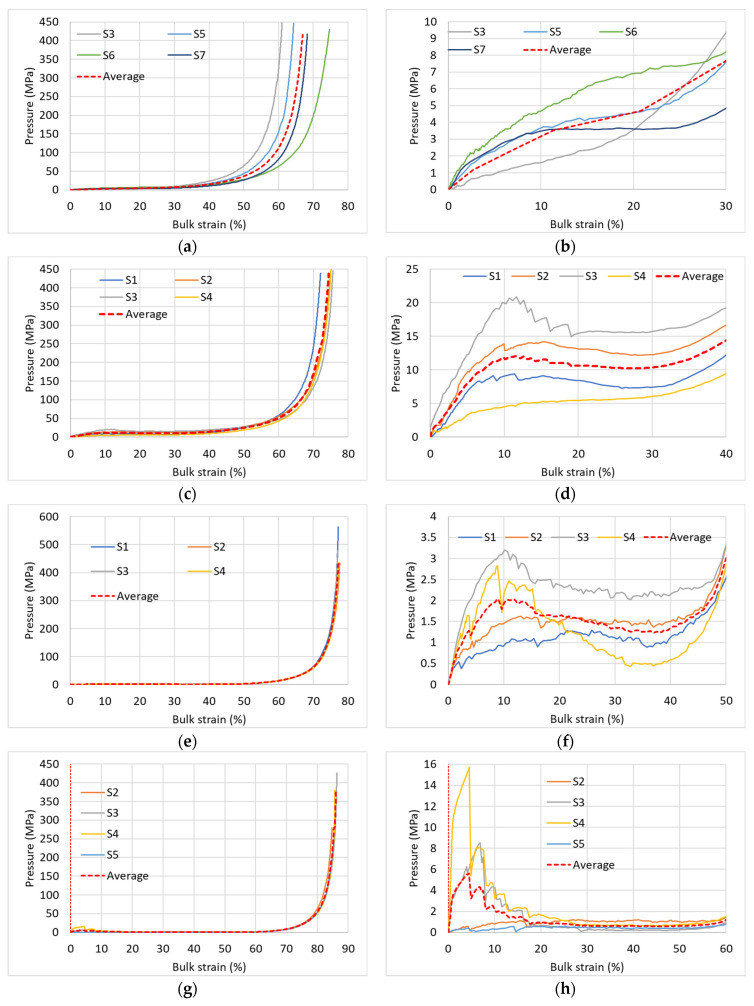
Repeatability. (**a**) D660—entire pressure range; (**b**) D660—low pressure (no “plateau”); (**c**) D600—entire pressure range; (**d**) D600—low pressure (“plateau”); (**e**) D400—entire pressure range; (**f**) D400—low pressure (“plateau”); (**g**) D280—entire pressure range; (**h**) D280—low pressure (“plateau”).

Figure 5 shows the pressure-bulk density relationships of four specimens for each of the specimen types. All specimens were wrapped around by a Teflon film [51], and were tested with rubber and Teflon discs on top and bottom of the specimen (Figure 3). The figure presents the equations of state for specific specimens (denoted by S1, S2, …, etc., as detailed in Table 1). The average curve of all the same AAC-type specimens is presented as well. Figure 5a,c,e,g (left hand side figures) shows the relationships for the full pressure range, and Figure 5b,d,f,h (right hand side figures) focuses on the low-pressure range.

**Figure 6:**
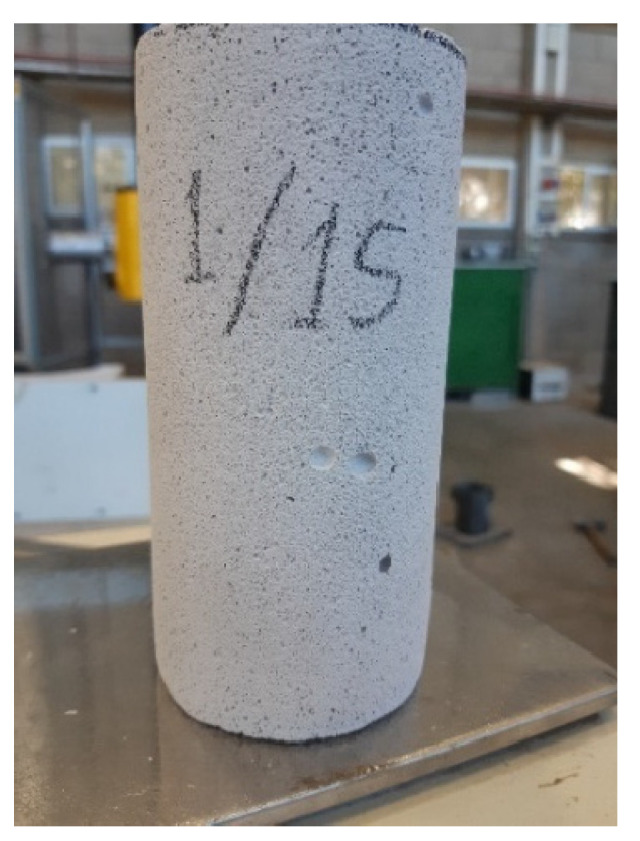
Discrete large pores in a highest density specimen.

The full range figures show excellent repeatability of the low density AAC specimens (D400 and D280), very good repeatability of the D600 density specimens, and reasonable repeatability with an observed scatter for the highest density D660 specimens. This worse repeatability of the high-density specimens may be explained by the fact that these specimens contain a limited number of relatively large pores (Figure 6), the number, size, and location of which vary from specimen to specimen and affect its non-uniform pressure-bulk strain behavior. However, even for this worse case of D660 specimens (Figure 5a), the repeatability may be considered satisfactory. The stiffnesses of these curves at the high-pressure range, corresponding to the stage at which most pores have already been closed, are similar. 

A closer look at the lower pressure range (Figure 5b,d,f,h) provides further new insights. Within that range, a considerable scatter is observed between the curves of different specimens having the same density. These curves may follow either a monotonic pressure increase accompanied with a stiffness increase, or a pressure increase with decreasing stiffness that is followed by a constant pressure plateau, or a pressure increase with a decreasing stiffness up to a local maximum pressure, followed by a decreasing pressure with increasing bulk strain until a local minimum pressure is reached that is followed by a pressure increase at larger strain. These entirely different curves have not been identified in earlier studies on AAC that are reported in the literature, showing a schematic line or an average single curve representing the behavior in that range [21,22,25,26,35,36]. It is important to note that the above inspected behavior has not been identified in our earlier studies on mortar and concrete. This is likely due to the considerably smaller pores in concrete and their characteristics. These preliminary results should be further investigated in a following research stage.

It may be concluded that in cases where the entire pressure range is of interest, the repeatability obtained from a limited number of specimens is satisfactory; however, if the lower pressure range is of interest, further research is required with a considerably larger number of specimens.

### 4.2. Effect of Boundary Conditions and Initial Cracking

In previous papers [50,51,52] dealing with cement paste, mortar, and concrete specimens, it had been shown that wrapping a specimen with a Teflon film was required to eliminate shear friction over the specimen boundary. Such friction stress adversely affects the specimen response, diminishes the major volumetric compression effect, and may cause premature shear fracture. In the present study, AAC porous specimens undergo considerably larger axial displacements, hence the boundary shear effect may be even more significant, and the specimens Teflon wrapping is even more important.

**Figure 7:**
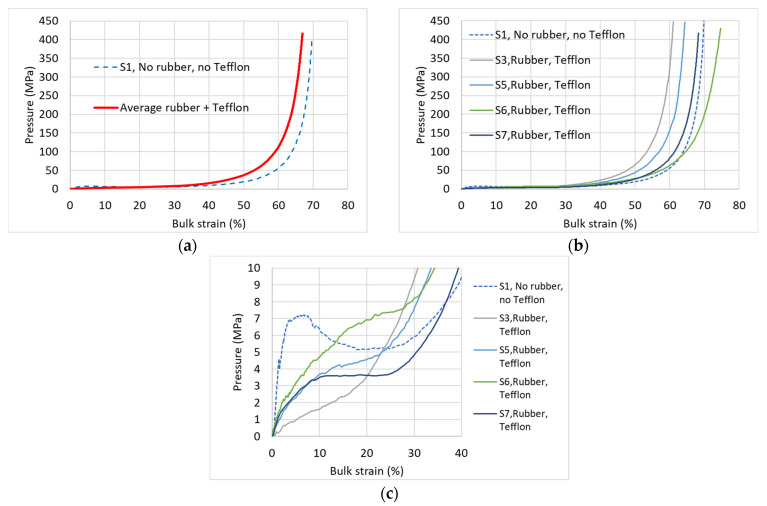
Effect of the rubber and Teflon discs—specimen D660. (**a**) Comparison with the average EOS; (**b**) comparison with all EOS—entire pressure range; (**c**) comparison with all EOS—low pressures.

Another important issue refers to the method of specimen loading. Concrete and mortar specimens are characterized by their relatively high stiffness and higher strength compared to AAC specimens. Therefore, in the case of concrete and mortar specimens the piston was directly loading the specimen top surface without causing any problem. In the present study of porous brittle AAC specimens that are characterized by considerably smaller stiffness and strength, application of the piston directly on the specimen’s top face may yield local crushing at the top and bottom contact surfaces. To examine this aspect, a special extra test has been conducted where a specimen, having the highest density D660, with the Teflon wrapping of the side surface (see Figure 3a), but excluding the rubber discs and the Teflon discs, was loaded. Figure 7a shows that, in this case, larger axial strain are developed at similar pressures, compared to the proposed “regular” setup where rubber and Teflon discs were included. This is clearly observed at the mid- and high-pressure levels (Figure 7b) showing the “No rubber, no Teflon” curve on the right side of the four “regular” specimens curves fan, and it is considerably different from their average (Figure 7a).

At the low pressure level (Figure 7c), focusing on the initial stage of the loading (up to ~8 MPa), a significant difference is observed between the “regular” four tested specimens and the present test (excluding rubber and Teflon): while the EOS of the ”regular” specimens show a gradual increase of the pressure-bulk modulus curve, and the curve stiffness decreases within the bulk strain up to ~30% where it stiffens up at larger strain, the present test shows a steep increase of the pressure-bulk strain curve to relatively considerably higher pressures, until a local maximum pressure is developed at a bulk strain of ~8% that is followed by a descent of the curve (negative stiffness) to a local minimum pressure at a bulk strain of ~25% and a following stiffening at larger strain that is similar to the trend of the four curves at that strain level.

**Figure 8:**
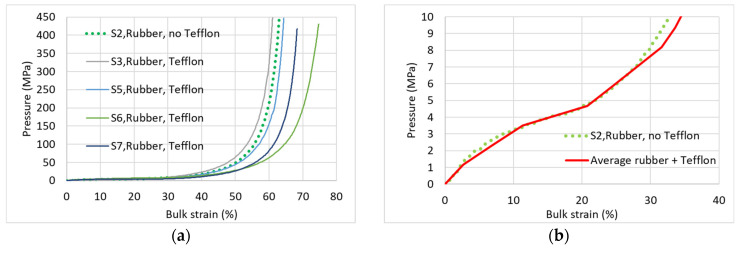

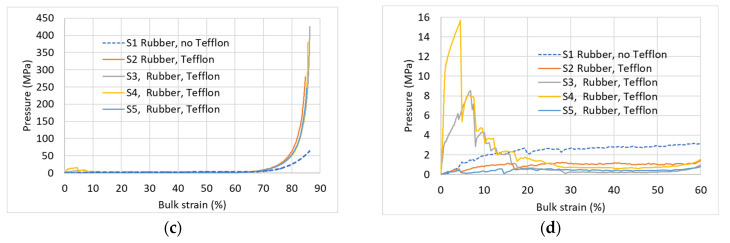
Effect of the Teflon disc. (**a**) D660—entire pressure range; (**b**) D660—low-pressure range; (**c**) D280—entire pressure range; (**d**) D280—low pressure range.

The effect of the intermediate 0.2-mm-thick Teflon discs that were placed under/above the rubber discs have been examined in extra tests carried out on specimens of the highest density (D660) and of the lowest density (D280). For the D660 specimen, no significant difference is identified (Figure 8a,b).

For the D280 specimen, the low pressure-bulk strain response starts similarly to the sparse distribution of the “regular” four curves, tested with rubber and Teflon discs (Figure 8d). However, for larger strain up to ~85% that are related to an axial deformation of ~108 mm (the maximum piston stroke), the D280 specimen does not stiffen up (dashed line, Figure 8c). The maximum force and corresponding hydrostatic pressure are 16.5 ton and 17.7 MPa, which are ~4% of the values (~400 ton and ~400 MPa) reached for specimens under “regular” test conditions.

**Figure 9:**
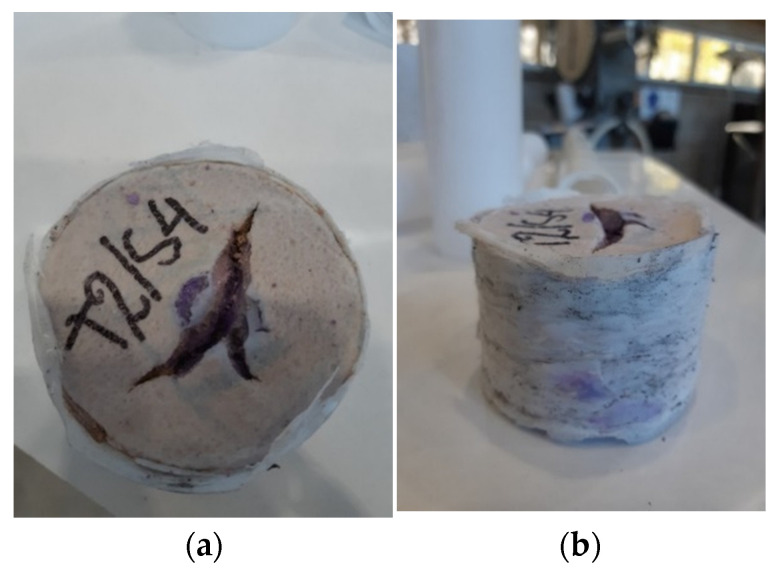
Damage identified after unloading specimen—D660. (**a**) top-view; (**b**) side-view.

**Figure 10:**
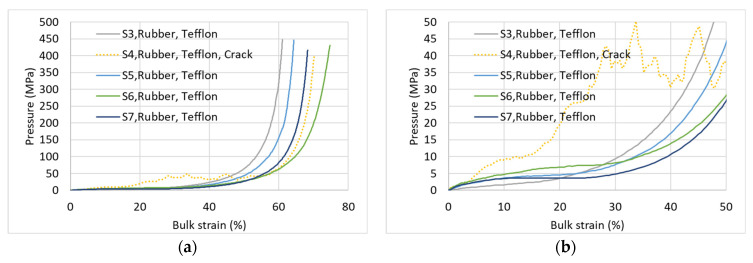
Effect of the initial crack. (**a**) Entire-pressure range; (**b**) low-pressure range.

After the unloading and piston removal, a peculiar damage was observed in one of the “regular” specimens with density D600 (tested with rubber, Teflon discs, and side wrapping). A major deep crack penetrating from the specimen top face into the specimen has been identified (Figure 9). Apparently, it indicates the existence of a relatively large interior crack in that specimen that has not been visible a priori. Figure 10b shows the significant effect of that damage on the initial segment of EOS, up to a bulk strain of ~50%, after which the curve stiffens up similarly to the four “regular” curves (Figure 10a).

### 4.3. Comparisons of Different AAC Densities

**Figure 11:**
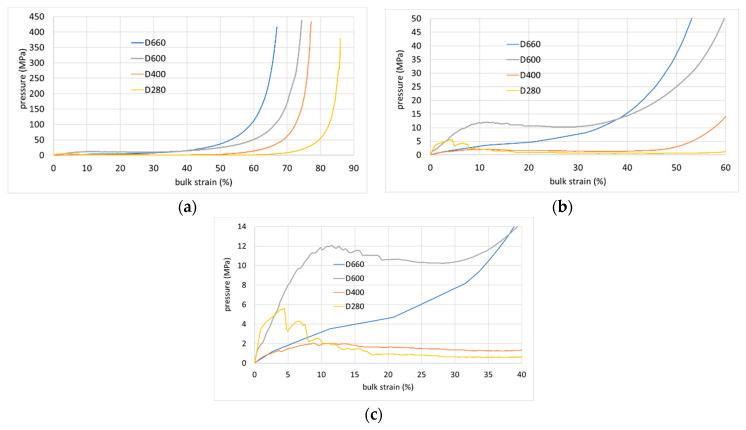
Average EOS curves for different AAC types. (**a**) Entire pressure range; (**b**) medium-pressure range; (**c**) low-pressure range.

Figure 11 compares average EOS curves of different AAC densities, which are presented in detail in Figure 5. Within the low-pressure range (Figure 11c) the EOS curve of the lower density specimens indicating specimens of higher total porosity (D400 and D280) exhibits a higher stiffness at smaller strain, and the curves climb to higher pressures compared to the curves related to higher densities. These curves reach a local maximum, after which the curve somewhat descends towards a local minimum pressure or a constant pressure over a rather large bulk strain range (”plateau”). At increasing bulk strain, the curves stiffen up sharply (Figure 11b,c). The ratio between this local peak and the plateau pressure level, as well as the plateau width (in terms of the bulk strain range), are larger for lower-density specimens (Table 2). The average EOS of specimens D600 reaches a local peak, after which the curve slightly descends to a local minimum pressure, which is followed by a ascending branch (Figure 11b,c).

The average EOS curve representing the specimens of highest density D660 increases monotonically (Figure 11b,c). The average EOS curve representing lower density specimens exhibits a local maximum at the EOS curve. Such local maximum had been observed in low density aluminum foam [58,59,60,61].

Similarly to the classification given for aluminum foams [58], the average EOS curves for AAC can be sorted out into following three classes:

- Brittle with significant disintegration, which have a clear local maximum prior to a pressure plateau.
- Ductile- with clear pressure plateau.
- Ductile without a pressure plateau.

### 4.4. Plastic Deformation and Energy Dissipation

**Figure 12:**
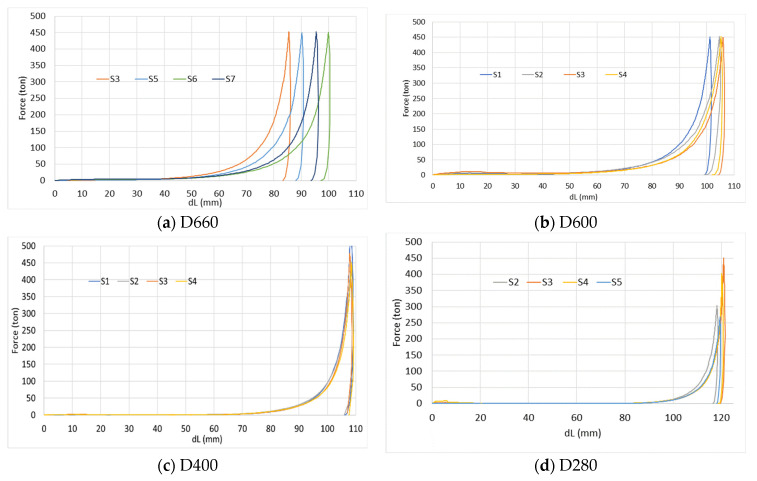
Force-specimen-shortening curves for different AAC types.

As may be seen from the previous analysis, deformation of AAC is characterized by significant plastic strain and crushing of the pores (especially large pores), and therefore it is accompanied by significant energy dissipation. This section provides a preliminary analysis of the total dissipation energy (i.e., the plastic deformation energy) that is based on the force-specimen shortening curves. These curves are presented in Figure 12. Note that, during the tests, both steel components and rubber discs behave elastically, and, therefore, do not absorb energy. 

The energy dissipation is calculated as an integral of the curve at three load levels (very low load level—150 kN, low load level—450 kN, and high load level—4500 kN). The results for each specimen of each AAC type are shown in Table 3. It shows that the scatter of results for different specimens of the same type (exception for D400) is very large for a very low load level (see Figure 11c), for which the covariance (COV) is larger than 20%. For a low-load-level load it is 12–15%, and for a high load level it is ~5–6% only. For D400, COV is 4–5.5% for all loads.

The “Average” column in Table 3 indicates an optimal AAC type (in the present study it is D600) that absorbs maximum energy during the pores crushing process for all examined load levels.

This is an interesting and important finding, indicating that there is an optimal density at which the largest energy may be dissipated under a given action. It stems from the fact that a low-density material is characterized by large displacement (deformation) at low resistance (pressure), and a high-density material is characterized by a relatively shorter deformation and a relatively higher resistance (pressure). The energy dissipation is related to the integrated value of the variable pressure over the range of displacements. It is likely that an intermediate value of density that is characterized by intermediate values of pressure and displacement will yield a maximum value of the integrated dissipation energy. This preliminary result should be further investigated along a more refined series of densities using a larger number of specimens.

## 5. Preliminary Thoughts on Impact Response of Different AAC Types

### 5.1. Preface

This paper’s objective is to experimentally investigate the EOS of AAC of different densities. Section 1, Section 2, Section 3 and Section 4 above presents the research conducted on the subject, including much new information and significant contribution to the state of the art. From the new EOS curves for different AAC densities, the dissipated energy was calculated, and an optimal density was found that is associated with maximum dissipated energy.

In this section, a preliminary study is presented, aiming at examination how different energy dissipation capacities of the different AAC types affects the impact response of the different AAC types. This is beyond the scope of the paper objective; nevertheless, it is tightly related to it. There are no theoretical/experimental studies available on this subject. Only the produced unique experimental data for the EOS of different AAC types enable a preliminary theoretical trial, and lack of test data prevents any validation. We were challenged to investigate this unexplored problem and confirm the relationship between the impact response and the energy dissipation capacity. This investigation should be continued, and, presently, no practical engineering conclusions and recommendations can be drawn.

### 5.2. Numerical Simulation of AAC under Impulsive Loading—Preliminary Analysis

**Figure 13:**
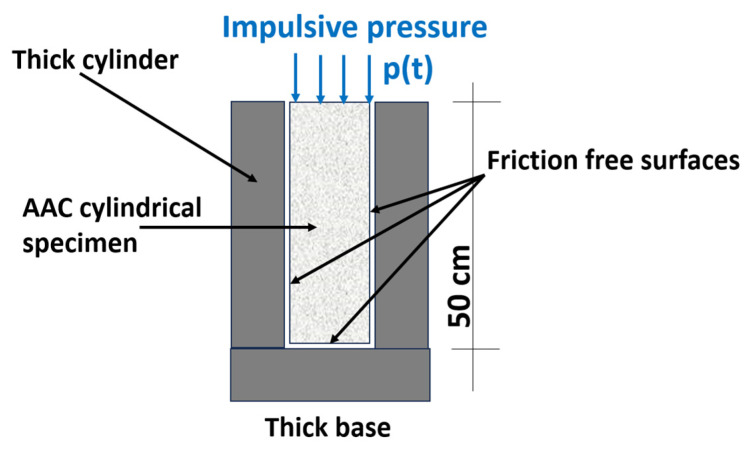
Scheme of the numerical problem.

It is conjectured that the different energy dissipation capacity of the different AAC types (Table 3) should affect their different dynamic response to a given dynamic load. To confirm that conjecture, a numerical simulation of a simple problem has been carried out. It refers to a 50 cm high ACC cylinder that is placed in a rigid cylinder, as shown in Figure 2a. The cylinder boundary is isolated to avoid friction with the cylinder interior boundary. The cylinder top surface is loaded by an evenly distributed impulsive load (Figure 13). Since such a test is extremely complicated to conduct, especially at very high pressures, a 1-D numerical simulation has been carried out using the same EOS curves, as determined above. For the numerical analysis, the AAC cylinder has been subdivided into 100 cells along its height (i.e., the cell size is 5 mm).

Considering the four different densities in Table 3, and using the corresponding EOS curves, dynamic analysis has been performed for the considered dynamic load corresponding to the force level where COV was relatively small for all AAC types (Table 3).

The dynamic load *p*(*t*) is described by a plane pressure wave having a following rectangular time dependence:

(5) $$p(t)=\left\{\begin{array}{c} p_{max},\;\;\;t\leq t_{D}\\ 0,\;\;\;t>t_{D} \end{array}\right.$$

where, (*p_max_* = 50 MPa, *t_D_* = 1 ms) and (*p_max_* = 100 MPa, *t_D_* = 0.5 ms) having the same impulse of 50 MPa∙ms.

**Figure 14:**
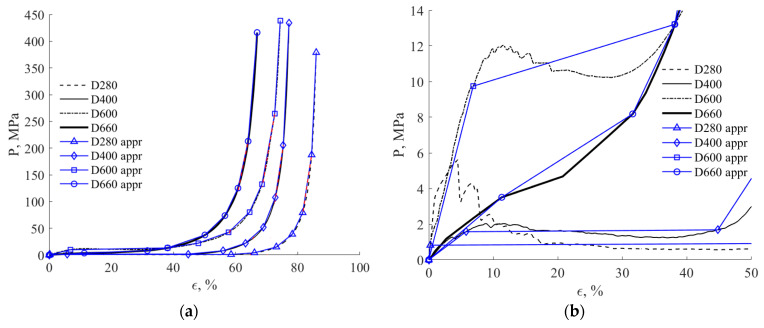
Approximated EOS for different AAC types. (**a**) Entire range of the pressure; (**b**) low pressures.

The analysis has been performed by explicit FEM in ANSYS AUTODYN commercial software [54] using a Lagrange approach. The behavior of the AAC cylinder was modeled in a hydrodynamic formulation using the “COMPACTION” equation of state provided by the ANSYS AUTODYN. For this model, the average experimental equations of state (see Figure 11) are approximated by 10 points, as shown in Figure 14. In Figure 14, the legend “appr” indicates the approximation curves, and the markers and the blue lines indicate the points of approximation and the approximation pricewise curves. The black lines present the measured in the test data. The notations of the AAC type (D280, D400, D600 and D660) correspond to those given in Table 1.

The system of the equations of motion of a continuous medium is of a hyperbolic type, and therefore a monotonic behavior of the EOS is required to ensure a positive sound velocity. In the present case (see Figure 11), only the EOS for D660 is monotonic, while the other three types (D600, D400, D280) have a local maximum at their initial segment (bulk strain less than 15%). Therefore, a procedure to turn these EOS curves to an approximate monotonic type is required. For that purpose, the approximated values at the first three points $(\varepsilon_{i},$ $p_{i}$) (*i* = 1, 2, 3) of the “COMPACTION” EOS were calculated using equivalence of the absorbed energy per unit volume:

(6) $$\int_{0}^{\varepsilon_{3}}p\left(\varepsilon \right)d\varepsilon =\int_{0}^{\varepsilon_{3}}p_{appr}\left(\varepsilon \right)d\varepsilon =\sum_{j=1}^{j=2}\left(\frac{p_{j}+p_{j+1}}{2}\right)\left(\varepsilon_{j+1}-\varepsilon_{j}\right)$$

where $\varepsilon_{1}=0$, $p_{1}=0$.

Here *p*(*e*) and *p_appr_*(*e*) are the measured and approximated EOS, respectively.

Approximated EOS curves are presented in Figure 14, and the relative error for the following seven points of the of EOS does not exceed 2%.

**Figure 15:**
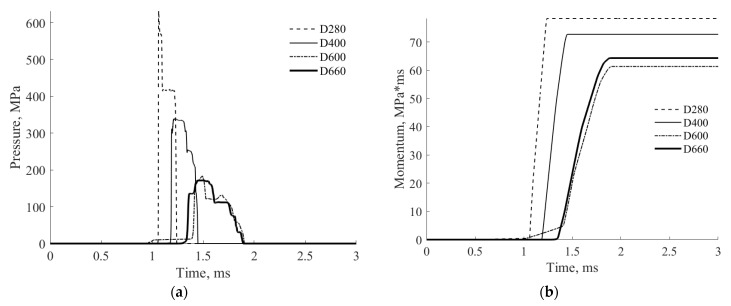

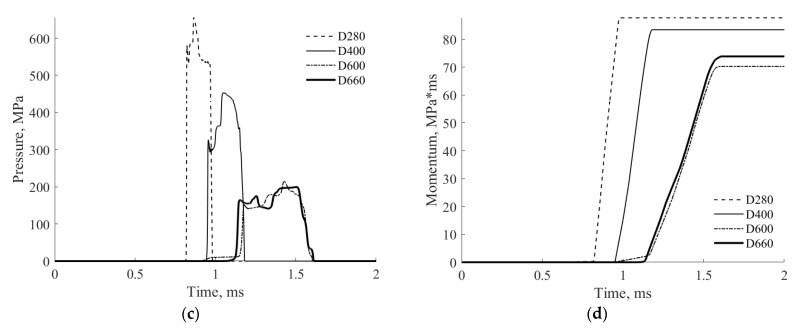
Time histories of the contact pressure and impulse. (**a**) Contact pressure, *p_max_* = 50 MPa, *t_D_* = 1 ms; (**b**) impulse, *p_max_* = 50 MPa, *t_D_* = 1 ms; (**c**) contact pressure, *p_max_* = 100 MPa, *t_D_* = 0.5 ms; (**d**) impulse, *p_max_* = 100 MPa, *t_D_* = 0.5 ms.

Figure 15 shows the contact pressures and impulses at the top face of the rigid cylinder, supporting the tested cylindrical specimen, for the considered four AAC types. 

Figure 15a,c shows that the contact peak pressure for D280 and D400 are larger in ~3 and ~1.5 times (respectively) than for denser AAC, D600, and D660, for which the peak pressures are approximately similar. However, the integrated pressure–time curve, which expresses the impulse, indicates that that the area under the curve corresponding to D600 is smaller than that corresponding to D660 for both loading levels, is smaller by~10% than the total impulse for D660, and considerably smaller than the total impulse for D280 and D400 AAC cylinders (Figure 15b,d).

The result of this dynamic analysis is in agreement with the conclusion (Table 3) regarding the energy dissipation based on the EOS curve, indicating that from the energy dissipation point of view, there exists an optimal type (density) of AAC. This density, (D600) as well as the corresponding optimal (maximum) energy values, are highlighted in bold in Table 3.

## 6. Conclusions

The present paper aims to experimentally study the EOS of autoclaved aerated concretes (AAC) with different densities, which has not been investigated before. Using a home-made high-pressure apparatus, oedometric tests of AAC specimens with different densities were performed. Due to the pronounced compressibility of these specimens under high pressures, and the resulting wide range of bulk strains, a special algorithm is proposed for the hoop strain calculations that is based on piecewise linear weighted functions. 

The effects of boundary conditions were eliminated by specimen wrapping with a Teflon film and by using rubber discs on the specimens’ top/bottom surfaces.

The repeatability of the results of four specimens of the same type showed better repeatability for low density compared to high density specimens. This is explained by the fact that, in higher density specimens, a smaller number of larger pores exist, thus affecting a nonuniform pore distribution. However, even for the worse case, good repeatability was observed. 

A significant scatter of the results has been identified at the low-pressure range that corresponds to the crushing of non-uniformly distributed pores. This scatter increases at lower densities. Therefore, testing at the low-pressure range requires more specimens to obtain a reliable mean EOS curve.

Comparison of average EOS curves for different types of AAC shows that, within the initial deformation range, the EOS curve of lower density specimens reach a local maximum, after which the curve somewhat “plateau” (or a local minimum), followed by a region of sharp strengthening. Other interesting features of the EOS curves have been identified and analyzed.

The novelty of this paper is comprises the following new contributions: providing the EOS curves for AAC of different densities; extending the common testing procedure to account for the pronounced compressibility occurring during testing; and providing insights on the early-stage damage processes and their effect on the shape of the EOS curve.

Lastly, an additional section, beyond the scope of this paper, has been added, aiming at analyzing the impact response of different AAC types. This has not been investigated earlier, and there are no test data available for validation. However, the present research yielded the EOS curves of different AAC types, and enabled a numerical preliminary investigation. The dissipation energy has been calculated for each specimen of each type of AAC. It was found that there exists an optimal AAC density that absorbs maximum energy. This is an interesting early finding that should be further investigated.

## Figures and Tables

**Table 1:** Specimen data.

Type	Specimen	Weight (g)	Height (mm)	Diameter (mm)	Density (kg/m^3^)		Density CoV (%)
					By Samples	Ave.	
D660	S1	353.2	138.27	68.70	689.13	663.95	2.59
	S2	336.2	140.43	68.17	655.98		
	S3	344.1	140.03	68.73	662.26		
	S4	345.1	140.13	68.50	668.24		
	S5	342.1	138.90	68.03	677.51		
	S6	323.5	139.67	68.17	634.67		
	S7	333.3	139.77	67.83	659.87		
D600	S1	312.6	140.40	69.00	595.43	601.17	0.84
	S2	310.8	139.63	68.80	598.72		
	S3	307.5	140.07	68.03	603.92		
	S4	308.1	140.13	67.93	606.59		
D400	S1	205.8	140.07	68.07	403.79	421.48	3.21
	S2	219.8	139.40	68.23	431.20		
	S3	208.6	140.00	67.37	418.03		
	S4	222.1	140.30	68.23	432.92		
D280	S1	147.6	140.03	67.97	290.52	290.24	1.43
	S2	144	140.00	67.87	284.34		
	S3	147.5	140.07	67.40	295.15		
	S4	147.3	140.20	68.10	288.45		
	S5	148.8	140.10	67.97	292.74		

**Table 2:** EOS plateau parameters.

Type	Plateau Pressure (MPa)	Peak-to-Plateau Pressures Ratio	Plateau Bulk Strain Range (%)
D280	0.6	10.2	15–60
D400	1.33	1.65	15–45
D600	10.6 (minimum value)	1.18	~0 (one point)
D660	no	no	no

**Table 3:** Energy dissipation (in KJ).

Force (kN)	S1	S2	S3	S4	Average	COV (%)
D280 *						
150	1.39	1.77	2.43	1.27	1.72	26.29
450	3.55	4.07	4.76	3.49	3.97	12.87
3000	14.72	15.24	15.19	13.50	14.66	4.80
D400						
150	1.61	1.84	1.83	1.73	1.75	5.35
450	4.31	4.81	4.68	4.73	4.63	4.13
4500	23.65	25.91	22.48	23.71	23.94	5.19
D600						
150	3.02	4.56	5.59	3.12	4.07	26.25
450	7.18	9.34	10.38	7.37	8.57	15.72
4500	36.72	43.32	39.57	37.32	39.23	6.59
D660						
150	1.89	2.20	3.05	3.12	2.56	20.82
450	5.63	5.91	7.54	7.32	6.60	12.73
4500	34.23	34.20	38.69	35.39	35.63	5.14

* D280 specimens with largest deformations were tested up to 3000 kN due to the piston stroke limit.

## Data Availability

Data are contained within the article.

## Nomenclature

AAC	Autoclaved Aerated Concrete
*D*_0_	Outer diameter of the device cylinder
*d*_0_	Specimen diameter
D280	AAC type with the density of 280–300 kg/m^3^
D400	AAC type with the density of 403–430 kg/m^3^
D600	AAC type with the density of 595–610 kg/m^3^
D660	AAC type with the density of 630–690 kg/m^3^
EOS	Equation of State
*F*	Applied force
*h_up_*	Distance of the upper-level’s SG from the top point of the device cylinder
*h_mid_*	Distance of the mid-level’s SG from the top point of the device cylinder
k	Stiffness of the “piston-rubbers-spacer” component of the device
LVDT	Linear Variable Displacement Transducers
*l*_0_	Specimen height
*p*	Hydrostatic pressure
*p_i_*, *i* = 1, 2, 3	Approximated pressure values at the first three points of the “COMPACTION” EOS
*p*(*t*)	Impulsive pressure
*p*(*e*)	Measured EOS
*p_appr_*(*e*)	Approximated EOS
*p_max_*	Value of the rectangular impulsive pressure
SG	Strain Gauge
*t_D_*	Duration of the rectangular impulsive pressure
*Dl*	Vertical displacement of the piston
*w_up_*	Weighting functions for measured hoop strain at the upper level
*w_mid_*	Weighting functions for measured hoop strain at the middle level
*w_down_*	Weighting functions for measured hoop strain at the lower level
$\varepsilon_{hoop}$	Hoop strain
$\varepsilon_{i},$ *i* = 1, 2, 3	Approximated strain values at the first three points of the “COMPACTION” EOS
*e_up_*	Averages of three SGs’ measurements at the upper levels
*e_mid_*	Averages of three SGs’ measurements at the middle levels
*e_down_*	Averages of three SGs’ measurements at the lower levels
*e_V_*	Bulk strain
$\varepsilon_{z}$	Axial strain
λ	Lateral pressure coefficient
$\sigma_{r}$	Lateral pressure
